# NLRP7 contributes to in vitro decidualization of endometrial stromal cells

**DOI:** 10.1186/s12958-017-0286-x

**Published:** 2017-08-15

**Authors:** Jyun-Yuan Huang, Pei-Hsiu Yu, Yueh-Chun Li, Pao-Lin Kuo

**Affiliations:** 1 Department of Obstetrics and Gynecology, National Cheng Kung University Hospital, 138 Sheng-Li Road, Tainan, 704 Taiwan; 2 Department of Biomedical Sciences, Chung Shan Medical University, No.110, Sec. 1, Jianguo N. Rd., South Dist, Taichung City, 402 Taiwan

**Keywords:** NLRP7, Endometrial stromal cells, Decidualization, Progesterone receptor

## Abstract

**Background:**

Nucleotide-binding oligomerization domain (NACHT), leucine rich repeat (LRR) and pyrin domain (PYD) 7 containing protein, NLRP7, is a member of the NLR family which serves as innate immune sensors. Mutations and genetic variants of *NLRP7* have been found in women with infertility associated conditions, such as recurrent hydatidiform mole, recurrent miscarriage, and preeclampsia. Decidualization of endometrial stromal cells is a hallmark of tissue remodeling to support embryo implantation and proper placental development. Given defective decidualization has been implicated in miscarriage as well as preeclampsia, we aimed to explore the link between the *NLRP7* gene and decidualization.

**Methods:**

Endometrial samples obtained from pregnant women in the first trimester and non-pregnant women were used to study NLRP7 expression pattern. The human telomerase reverse transcriptase (hTERT)-immortalized human endometrial stromal cells (T-HESCs) were used to study the effect of NLRP7 on decidualization. Decidualization of T-HESCs was induced with 1 μM medroxyprogesterone acetate (MPA) and 0.5 mM 8-bromoadenosine 3':5'-cyclic monophosphate (8-Br-cAMP). siRNA was used to knock down NLRP7 while lentiviral vectors were used to overexpress NLRP7 in cells. NLRP7 expression was detected by immunofluorescence, qRT-PCR, and Western blotting. Decidualization markers, Insulin-like growth factor-binding protein 1 (IGFBP-1) and prolactin (PRL), were detected by qRT-PCR and ELISA. Nuclear translocation of NLRP7 was detected by the subcellular fractionation and confocal microscopy. The effect of NLRP7 on progesterone receptor (PR) activity was evaluated by a reporter system.

**Results:**

NLRP7 was up-regulated in the decidual stromal cells of human first-trimester endometrium. After in vitro decidualization, T-HESCs presented with the swollen phenotype and increased expressions of IGFBP-1 and PRL. Knockdown or over-expression of NLRP7 reduced or enhanced the decidualization, respectively, according to the expression level of IGFBP-1. NLRP7 was found to translocate in the nucleus of decidualized T-HESCs and able to promote PR activity.

**Conclusions:**

NLRP7 was upregulated and translocated to the nucleus of the endometrial stromal cells in an in vitro decidualization model. Overexpressed NLRP7 promoted the IGFBP-1 expression and PR reporter activation. IGFBP-1 expression decreased with the knockdown of NLRP7. Therefore, we suggest that NLRP7 contributes to in vitro decidualization of endometrial stromal cells.

## Background

Nucleotide-binding oligomerization domain (NACHT), leucine rich repeat (LRR) and pyrin domain (PYD) 7 containing protein, NLRP7, is a member of the NLR family which serves as intracellular sensors of innate immunity to regulate inflammation and cell apoptosis [1]. The *NLRP7* transcripts have been identified in a large number of human tissues, including liver, lung, placenta, spleen, thymus, peripheral blood leukocytes, testis, and ovaries [2]. NLRP7 has a well-studied role in regulating immune responses [3–5]. In 2006, the *NLRP7* gene was also identified as a maternal locus associated with recurrent hydatidiform mole (HM), recurrent miscarriage (RM), and recurrent preeclampsia [6]. Since then, many groups have identified mutations as well as genetic variants of *NLRP7* in women who experienced recurrent HM and RM [7–10]. HM is an abnormal human pregnancy with no embryo and cystic degeneration of placental villi. It is an imprinting disorder caused by lack of maternally acquired DNA methylation at germline differentially methylated regions [11, 12]. A report showed that NLRP7 protein affects trophoblast lineage differentiation by interacting with YY1 protein within the nucleus to alter gene methylation in human embryonic stem cells [13]. *NLRP7* is thus a maternal-effect gene involved in imprinting acquisition in the oocyte [14, 15].

RM is the occurrence of at least two consecutive early pregnancy losses. Various factors have been identified for RM, such as impaired decidualization of the endometrium, uterine anomaly, chromosomal abnormalities, endocrine dysfunction, thrombophilia, immune disorders, lifestyle factors and maternal infections. In up to 50% of cases, the cause of RM remains undetermined [16–18]. A study showed that women who experienced RM without HM have non-synonymous variants of *NLRP7* [19]. We also found the *NLRP7* gene to be significantly associated with RM [20]. These findings suggest that HM and RM may share the same genetic etiology in some cases, and *NLRP7* is a strong candidate gene in this context.

In humans, the rising progesterone level after ovulation induces the decidualization of stromal cells, which transform from fibroblast-like cells into epitheloid-like cells and secrete a variety of phenotypic antigens, such as IGFBP-1 and PRL [21]. The decidualized stromal cells acquire unique biochemical and cellular properties to prepare a receptive environment for the implantation of the developing embryo, proper placentation, and pregnancy maintenance [22, 23]. Given defective decidualization has been implicated in miscarriage and preeclampsia [24, 25], in this study we sought to explore the link between the *NLRP7* gene and decidual function using an in vitro decidualization model.

## Methods

### Reagents

Rabbit anti-human NLRP7 antibody (IMG-6357A) was obtained from Novus Biologicals (Littleton, CO). Rabbit anti-human Lamin B antibody (sc-6216-R), mouse anti-human α-tubulin antibody (sc-5286), and mouse anti-human β-actin antibody (sc-47,778) were procured from Santa Cruz Biotechnology (Santa Cruz, CA). HRP-conjugated goat anti-rabbit antibody (GTX77060) and HRP-conjugated goat anti-mouse antibody (GTX213111–01) were acquired from GeneTex (Irvine, CA). A Human IGFBP-1 DuoSet ELISA kit (DY871) and normal goat serum (DY005) were purchased from R&D Systems (Minneapolis, MN). Antibody diluent with background reducing component (S3022) was procured from Agilent Technologies (Santa Clara, CA). Alexa Fluor 488-conjugated goat anti-rabbit antibody (A11008), Subcellular Protein Fractionation kit (78840), and Fast SYBR Green Master Mix (4385612) were obtained from ThermoFisher Scientific (Waltham, MA). The 3-Amino-9-ethylcarbazole (AEC) Chromogen/Substrate Bulk Kit (ACJ500) was purchased from ScyTek Laboratories (Logan, UT). MPA (M1629), 8-Br-cAMP (B5386), and mounting medium Glycerol Gelatin (GG1) for immunohistochemistry were procured from Sigma-Aldrich (St. Louis, MO). Mounting medium DAPI Fluoromount-G (0100–20) for immunofluorescence was obtained from SouthernBiotech (Birmingham, AL). Human NLRP7 full-length cDNA clone (SC323251) was obtained from OriGene. Lentiviral vectors pLAS2w.RFP-C.Pneo and pLAS2w.Pneo were procured from the National RNAi Core Facility, Academia Sinica (Taipei, Taiwan). A Cignal™ progesterone receptor (PR) reporter kit (CCS-6043 L) was acquired from QIAGEN (Germantown, MD). The Dual-Glo Luciferase assay system (E2920) was purchased from Promega (Madison, WI). A Direct-zol RNA MiniPrep kit (R2050) was acquired from Zymo Research (Irvine, CA).

### Cells

The T-HESCs were purchased from ATCC (CRL-4003TM). T-HESCs were cultured in DMEM/F12 medium without phenol red (D2906, Sigma) supplemented with 10% charcoal/dextran treated fetal bovine serum (SH30068.03, HyClone), 1.5 g/L sodium bicarbonate, 1% ITS+ Premix (354,352, BD), and 500 ng/mL puromycin. T-HESCs were grown in an incubator at 37 °C under a 5% CO2 atmosphere at constant humidity. To generate an in vitro decidualization model, T-HESCs were cultured in 2% charcoal/dextran treated FBS medium with or without 1 μM MPA and 0.5 mM 8-Br-cAMP [26]. After three days, the culture medium was renewed with the same treatment. On day 6, the decidualization was confirmed by observing the enlarged rounded cell shape under a microscope and measuring the expressions of IGFBP-1 and PRL by quantitative reverse-transcription PCR (qRT-PCR) or the IGFBP-1 protein in the supernatant by ELISA.

### Quantitative reverse-transcription PCR (qRT-PCR)

RNA samples of T-HESCs treated with or without MPA and 8-Br-cAMP were isolated by the Direct-zol RNA MiniPrep kit, with 2 μg of each sample being subjected to reverse transcription into cDNA. The expressions of IGFBP-1, PRL, and NLRP7, as well as glyceraldehyde 3-phosphate dehydrogenase (GAPDH), were quantified using a StepOnePlus real-time PCR apparatus (Thermo Fisher Scientific, LA, USA). Primers used for this study included: PRL forward primer: 5′ TCATCTGGTCACGGAAGTACGT 3′; PRL reverse primer: 5′ GCCCTCTAGAAGCCGTTTGG 3′; IGFBP-1 forward primer: 5′ ATGGCTCGAAGGCTCTCCAT 3′; IGFBP-1 reverse primer: 5′ TCCTGTGCCTTGGCTAAACTC 3′; NLRP7 forward primer: 5′ CTTCTGTGCGGATTCTTTGTGA 3′; NLRP7 reverse primer: 5′ TTTTTAATCTCCACTTTCTGCAGATG 3′; GAPDH forward primer: 5′ TGAAGGTCGGAGTCAACGGATT 3′; GAPDH reverse primer: 5’CCTGGAAGATGGTGATGGGATT 3′. PRL and IGFBP-1 primers were designed by using Primer Express® Software for Real-Time PCR (Version 3.0) from Applied Biosystems. NLRP7 and GAPDH primers were obtained from the literature [27, 28]. The qRT-PCR was performed using Fast SYBR Green Master Mix following the manufacturer’s instructions. Briefly, the PCR conditions were as follows: Step 1: 95 °C for 20 s for enzyme activation; and, Step 2: 40 cycles consisting of 95 °C for 3 s for denaturing, and 60 °C for 30 s for annealing/extending. A melt curve analysis was carried out on the products of the amplification reaction to ascertain the melting temperatures of these. The conditions were as follows: Step 1: 95 °C for 15 s; Step 2: 60 °C for 1 min; and, Step 3: 95 °C for 15 s. The amplicons were detected and quantified using SYBR Green dye. StepOne Software v2.3 (Applied Biosystems) was used to quantify the levels of expressions.

### Western blotting

The cell lysates were collected from T-HESCs treated with or without MPA and 8-Br-cAMP. The subcellular protein fractions were extracted by using the Subcellular Protein Fractionation kit. The total cell lysates or subcellular protein fractions were combined with 6X protein loading buffer (*v*/v = 5:1) and denatured in boiled water for 10 min. The treated cell lysates were then subjected to SDS-PAGE and thereafter transferred to PVDF membranes. After blocking, the membranes were incubated with the different primary antibodies, such as NLRP7, Lamin B, α-tubulin and β-actin at 4 °C overnight. Lamin B and α-tubulin were used as the nuclear marker and the cytosol marker, respectively. Bands were visualized using peroxidase-conjugated goat anti-mouse IgG and ECL plus reagents.

### ELISA

The supernatants of T-HESCs treated with or without MPA and 8-Br-cAMP were collected for detecting the IGFBP-1 protein by ELISA. The analysis was performed according to the manufacturers’ protocols. The cytokine productions were normalized according to the concentrations of cell lysate of the adhered cells, which were mostly alive.

### Immunohistochemistry

Endometrial samples were obtained from five legal abortions of first trimester pregnancies (between 8th and 12th week of gestation) with the permission of the ethical committee of the National Cheng Kung University Hospital. Endometrial samples obtained from five non-pregnant women who underwent diagnostic hysteroscopy served as the control. Donors signed an informed consent form approved by the Institutional Review Board of National Cheng Kung University Hospital (Tainan, Taiwan, Republic of China). The 4-μm tissue sections of the paraffin-embedded tissue underwent deparaffinization and rehydration. After blocking endogenous peroxidase activity by hydrogen peroxidase, the tissue sections were incubated with 5% normal goat serum in Tris-buffered saline, 0.05% Tween 20 (TBST) for 1 h. The tissue sections were then incubated with anti-human NLRP7 antibody diluted by the antibody diluent (1:50) at 4 °C overnight. After washing with the TBST, the tissue sections were incubated with HRP-conjugated goat anti-rabbit antibody diluted by the antibody diluent (1:200) at room temperature for 1 h. After washing with the TBST, the AEC substrate was added on the tissue sections. When red signals were observed, the tissue sections were washed with water and stained with the counter stain hematoxylin. The tissue sections were mounted using glycerol gelatin and observed with a microscope.

### Immunofluorescence

T-HESCs were cultured on coverslip and treated with or without MPA and 8-bromo-cAMP on day 0 and day 3. on day 6, the cells were fixed and permeabilized using 3.7% formaldehyde and 0.5% Triton X-100, respectively. After blocking in 5% normal goat serum in TBST for 1 h, the cells were treated with anti-human NLRP7 rabbit antibody diluted by the antibody diluent (1:50) at 4 °C overnight. After washing, the cells were incubated with Alexa Fluor 488 conjugated anti-rabbit antibody diluted by the antibody diluent (1:400) at room temperature for 1 h. After washing with the TBST, the cells were mounted using the DAPI Fluoromount-G and the signals of NLRP7 and DAPI were observed using a confocal fluorescence microscope (FV1000, Olympus).

### siRNA transfection and lentiviral infection

To down-regulate NLRP7 expression in T-HESCs, NLRP7 siRNA, 5′ GATGGCAAGAAACTGGCAGAA 3′ and negative control siRNA, 5′ UUCUCCGAACGUGUCACGUTT 3′ were used. T-HESCs were transfected with NLRP7 siRNA, denoted as T-HESCs (siNLRP7), and treated with or without MPA and 8-Br-cAMP on the same day. T-HESCs transfected with negative control siRNA, denoted as T-HESCs (siCtrl), served as the control group. On day 3, T-HESCs (siNLRP7) and T-HESCs (siCtrl) were transfected again with NLRP7 siRNA and control siRNA, respectively, and then treated with or without MPA and 8-Br-cAMP. On day 6, the cell pellets were collected for analyzing the expressions of NLRP7 and the supernatants were collected for detecting the expression of IGFBP-1.

The lentivirus system was used to up-regulate NLRP7 expression. The NLRP7 cDNA fragment was cloned into pLAS2w.Pneo vector to construct the NLRP7 expression vector. The pLAS2w.RFP-C.Pneo vector is a RFP expression vector that served as a control. The vectors were sent to the RNAi Core Facility of National Cheng Kung University Hospital to generate the NLRP7-expressed lentivirus (LV-NLRP7) and control RFP-expressed lentivirus (LV-RFP). Then, T-HESCs were infected with LV-NLRP7 or LV-RFP overnight and then selected by puromycin (0.5 μg/ml) for two weeks. The lentivirus-infected T-HESCs were treated with or without MPA and 8-Br-cAMP simultaneously. On day 6, the cell pellets were collected to analyze the expression of NLRP7 and the supernatants were collected to detect the expression of IGFBP-1.

### PR activity assay

The Cignal™ PR reporter kit was used to measure the activity of progesterone receptor-induced signal transduction pathways. The PR reporter is a mixture of a PR-responsive luciferase construct and a constitutively expressing Renilla element (40:1). The control reporter is a mixture of a non-inducible firefly luciferase construct and constitutively expressing Renilla luciferase construct (40:1). T-HESCs cultured in 96-well plates (1 × 10^4^ cells/well) were co-transfected with 100 ng PR reporter and 100 ng NLRP7-expressed vector or 100 ng RFP-expressed vector for 24 h. The cells co-transfected with the control vectors and NLRP7-expressed vector or RFP-expressed vector served as the negative control. After transfection, these cells were treated with 1 μM MPA and 0.5 mM 8-Br-cAMP for 24 h. The firefly and Renilla luminescence of the transfected cells were measure using a Dual-Glo Luciferase assay system (Promega, E2920) and plate-reading Luminometer (CentroPRO LB 962, Berthold Technologies). The ratio of firefly:Renilla luminescence in each well was calculated. The ratios of the PR reporter-transfected wells were normalized to those of the negative control reporter-transfected wells.

### Statistics

The results of ELISA and qRT-PCR are represented as means ±SD. Differences between groups were assessed by ANVOA, with a *p*-value less than 0.05 considered statistically significant.

## Results

### NLRP7 was expressed in the decidualized stromal cells

**Fig. 1:**
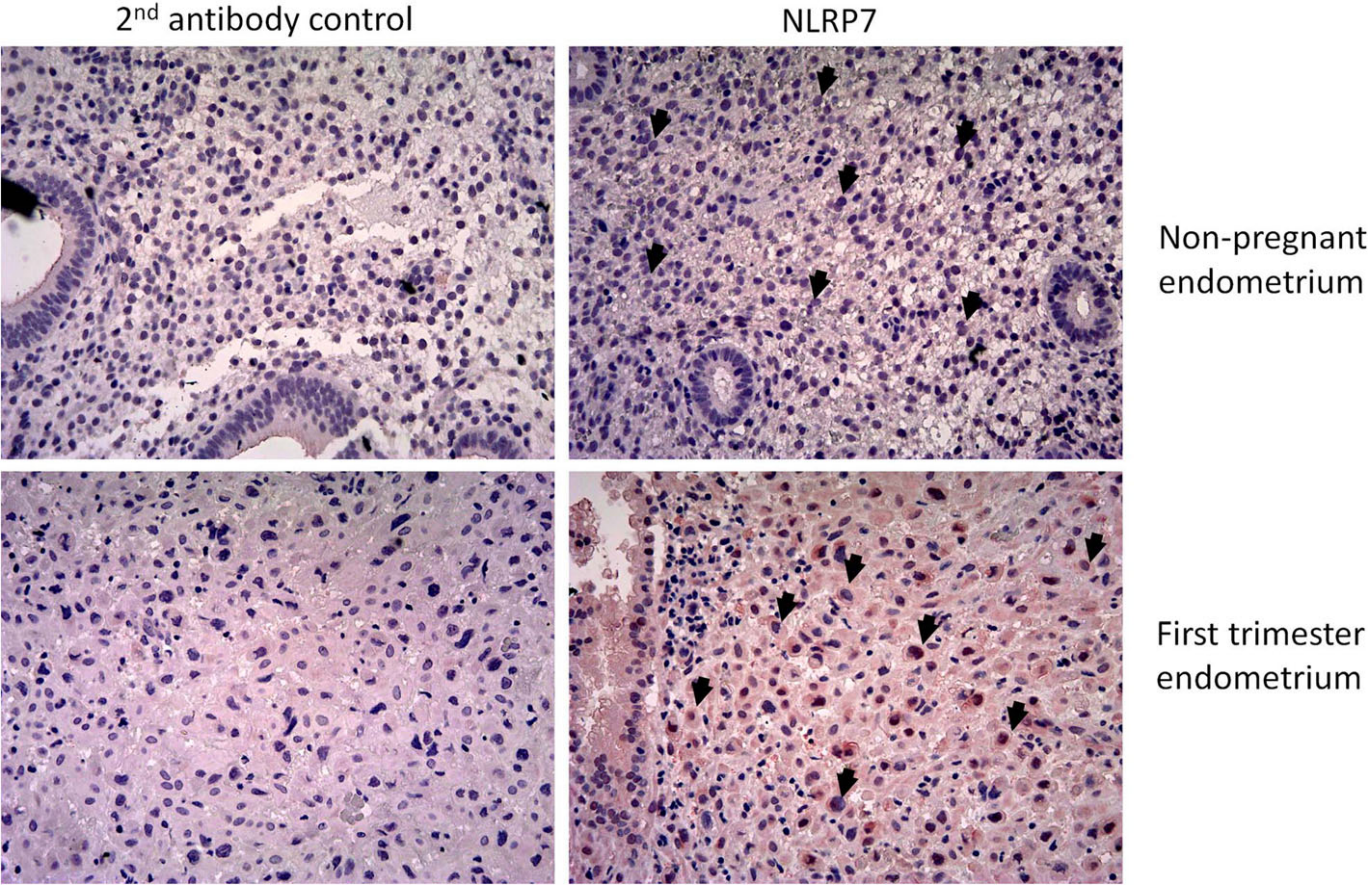
NLRP7 expressed in the decidualized stromal cells of the human endometrium during the first trimester. The tissue sections of the non-pregnant endometrium (*n* = 5) or the first trimester endometrium (*n* = 5) were deparaffinized, rehydrated and stained with NLRP7 antibody. Representative images of NLRP7 immunohistochemistry in the endometrium are shown. The NLRP7 signal was developed with the anti-rabbit HRP antibody and AEC substrate. Staining with the 2nd antibody only served as the negative control. Arrows point to the endometrial stromal cells. NLRP7 dominantly appeared in the swollen decidualized stromal cells of pregnant endometrium, but not in the stromal cells of non-pregnant endometrium (magnification 200X)

We aimed to explore the role of NLRP7 in early pregnancy. The NLRP7 expression in the endometrium of non-pregnant women and pregnant women during the first trimester was detected by immunohistochemistry. The NLRP7 protein was observed mainly in the swollen decidualized stromal cells of the endometrium samples of pregnant women. Many swollen decidualized stromal cells had NLRP7 protein expression in the cytosol as well as nucleus. NLRP7 protein was less expressed in the endometrial cells of non-pregnant women (Fig. 1).

**Fig. 2:**
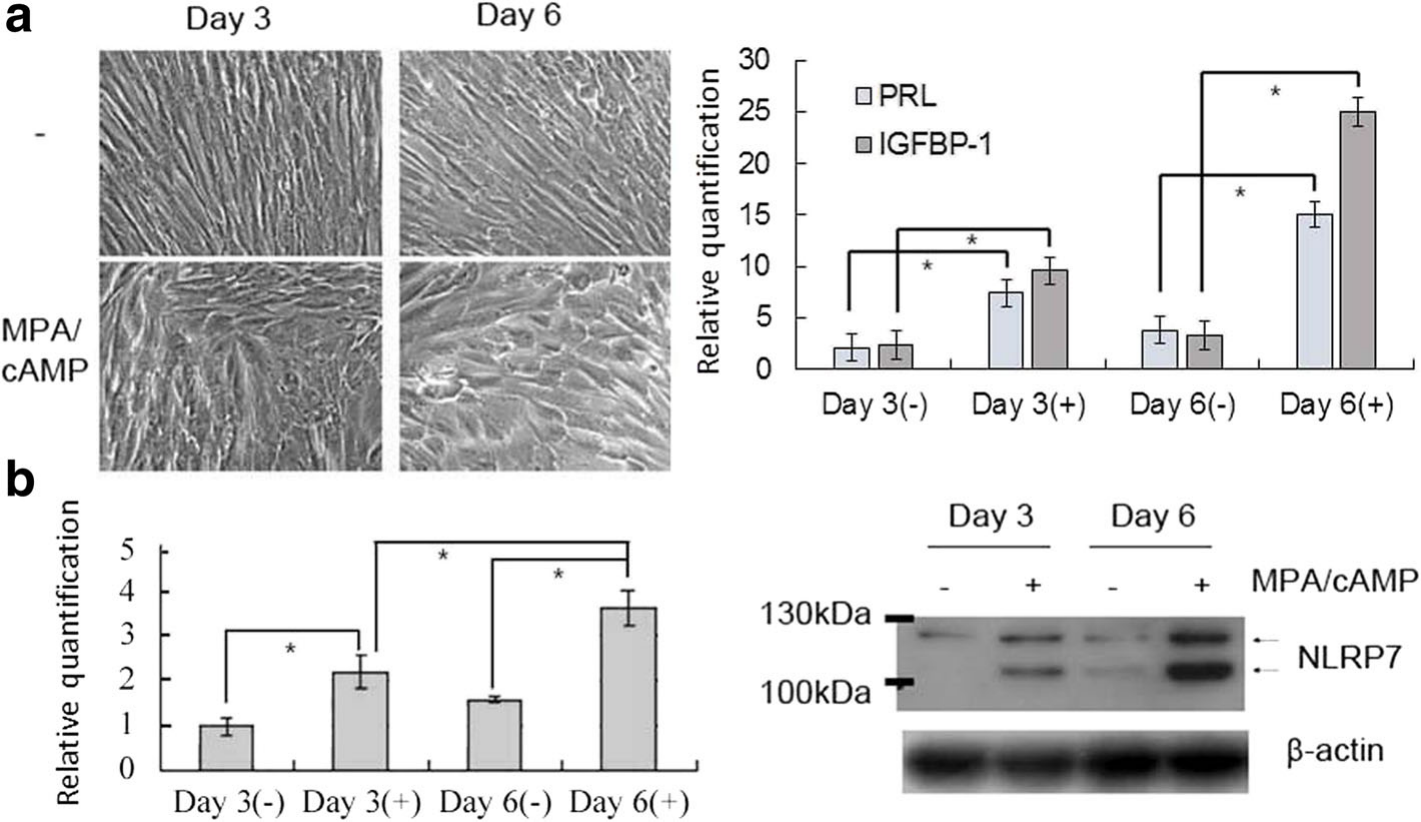
Increasing NLRP7 expression with in-vitro decidualization of T-HESCs. T-HESCs were treated with 1 μM MPA and 0.5 mM 8-Br-cAMP on day 0 and day 3 to induce decidualization. Untreated cells served as the control. **a** The cell morphology was evaluated using microscopy. The *PRL* and *IGFBP-1* transcripts were detected by qRT-PCR on day 3 and day 6. The treated cells displayed a swollen phenotype and produced more transcripts of two decidual markers, compared with the untreated cells. **b** The *NLRP7* transcript and protein in the treated or untreated cells was detected by qRT-PCR and Western blotting, respectively, on day 3 and day 6. The *NLRP7* transcript amount is shown in fold changes. Both *NLRP7* transcript amount and protein level were significantly higher in treated cells than in untreated cells. In the treated cells, the *NLRP7* transcript and protein level were also higher on day 6 than on day 3. The expressions of both NLRP7 isoforms were higher in the treated cells. The statistical differences were calculated from three independent experiments. *p*-values less than 0.05 are marked with “*”

To explore whether NLRP7 is involved in the decidualization pathways, we used an in vitro decidualization model of T-HESCs by treating the cells with progesterone analog MPA and 8-bromo-cAMP. The morphology of the treated cells changed from fibroblast-like into epithelioid-like on day 3 and day 6 (Fig. 2a left panel). The transcript amounts of the two decidualization markers, PRL and IGFBP-1, increased gradually in the treated cells compared to the untreated cells (Fig. 2a right panel). The NLRP7 transcript amounts were significantly increased after in vitro decidualization on day 3 and day 6, with the day-6 transcript amount being higher than that on day-3 (Fig. 2b, left panel). The results of the Western blot analysis show that the NLRP7 protein had two isoforms. The NLRP7 protein expression increased after in vitro decidualization, with the day 6 level being higher than that of day 3 (Fig. 2b, right panel). The short isoform (MW ~105 kDa) of NLRP7 increased to a greater extent than the long isoform (MW ~118 kDa) on day 6 after decidualization [29]. These results show that NLRP7 was up-regulated gradually with in vitro decidualized T-HESCs.

### NLRP7 expression level is related to in vitro decidualization

**Fig. 3:**
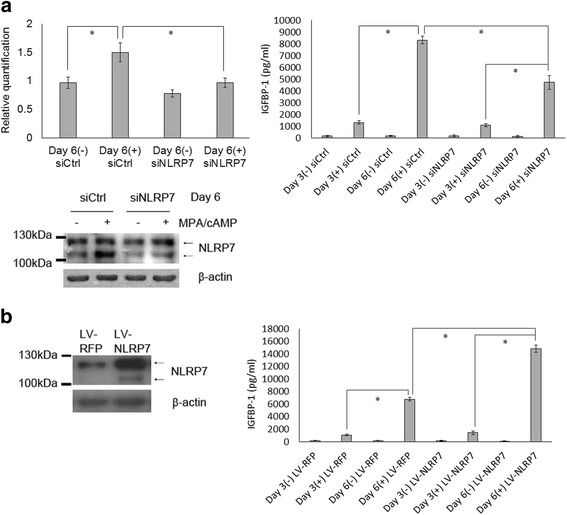
The expression level of NLRP7 is positively correlated with the decidualization degree of T-HESCs. **a** T-HESCs transfected with siNLRP7 or negative siCtrl were subjected to in-vitro decidualization. The *NLRP7* transcript and protein were analyzed on day 6. The total transcript as well as the short isoform of NLRP7 protein was suppressed by siRNA. Less abundant IGFBP-1 was produced by T-HESCs (siNLRP7), compared to T-HESCs (siCtrl). **b** T-HESCs infected with LV-NLRP7 or LV-RFP were subjected to in-vitro decidualization. T-HESCs (LV-NLRP7) had a significantly higher expression level of NLRP7 than T-HESCs (LV-RFP), as detected by Western blotting. The IGFBP-1 level was significantly higher in T-HESCs (LV-NLRP7), compared to T-HESCs (LV-RFP). The statistics were calculated from three individual experiments. *p*-values less than 0.05 are marked with “*”

Since NLRP7 was induced during the decidualization, we further explored the role of NLRP7 in the decidualization process using siRNA knockdown of NLRP7 and transfection of the exogenous NLRP7 full-length cDNA experiments. In the siRNA knockdown of NLRP7 experiment, the transcript and protein amount of NLRP7, compared with T-HESCs (siCtrl), decreased on day 6 after in vitro decidualization (Fig. 3a, left panel) (Fig. 3a, left panel). In particular, the short isoform of NLRP7 was significantly knocked down by the siRNA after in vitro decidualization (Fig. 3a, left panel). The cell decidualization was evaluated by the expression level of IGFBP-1 using ELISA. On day 6, the IGFBP-1 level of T-HESCs (siNLRP7) was significantly lower than that of T-HESCs (siCtrl) (Fig. 3a, right panel). This result of siRNA knockdown implies that the short isoform of NLRP7 was involved in the in vitro decidualization of T-HESCs.

In the expression of the exogenous NLRP7 full-length cDNA experiment, the protein expression of NLRP7 was more abundant in T-HESCs (LV-NLRP7) than in T-HESCs (LV-RFP) on day 6 after in vitro decidualization (Fig. 3b, left panel), confirming the expression of the transfected exogenous NLRP7 full-length cDNA via the lentivirus system. The increased NLRP7 proteins were mainly the large isoform of NLRP7. We also found that the IGFBP-1 level was significantly higher in the T-HESCs (LV-NLRP7) than in the T-HESCs (LV-RFP) on day 6 after in vitro decidualization (Fig. 3b, right panel). The results show that NLRP7 expression was related to the IGFBP-1 expression. Taking the results of knockdown and overexpression of NLRP7 together, it could be suggested that NLRP7 plays a role in the decidualization of T-HESCs.

### NLRP7 protein was translocated to the nucleus after decidualization

**Fig. 4:**
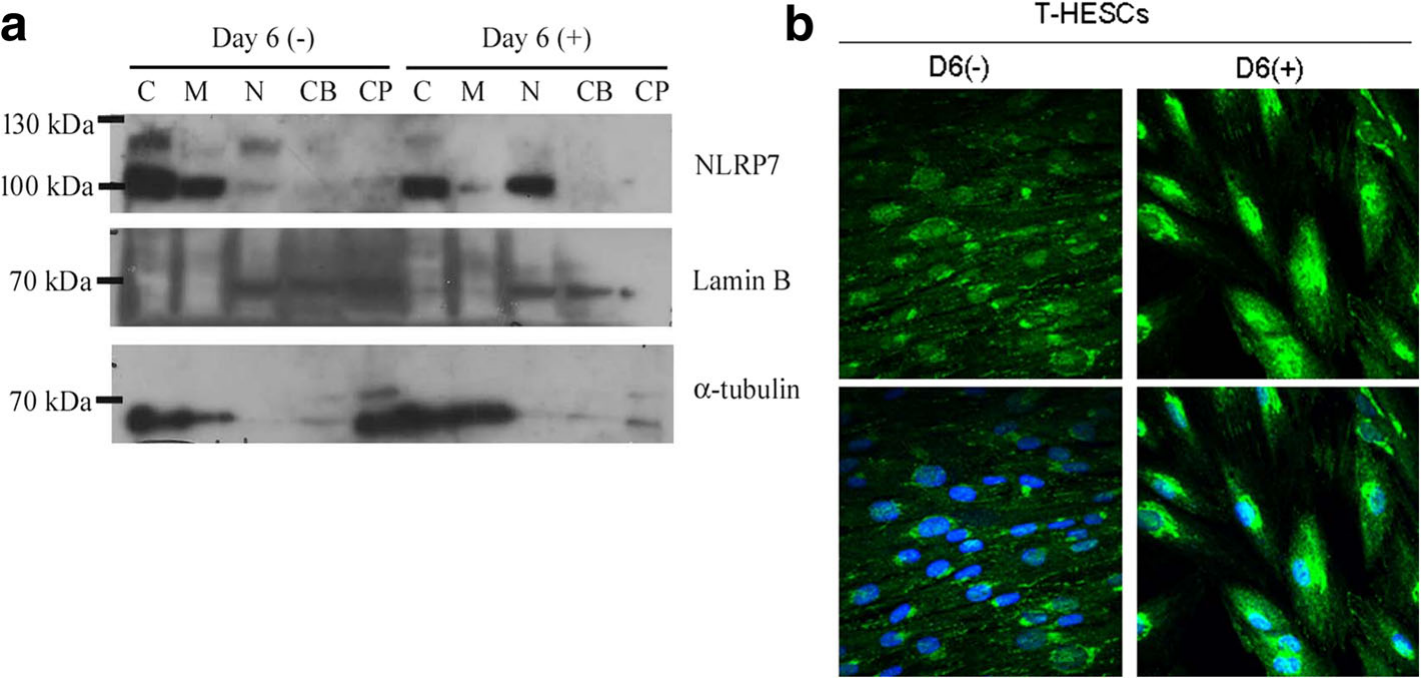
The location of NLRP7 protein in the decidualized human endometrial stromal cells. **a** The proteins of NLRP7, Lamin B, and α-tubulin in different subcellular fractions were analyzed by Western blotting. The short isoform of NLRP7 protein abundantly presents in the nuclear fraction after decidualization is induced. C: cytoplasmic protein; M: membrane protein; N: nuclear soluble protein; CB: chromatin-bound protein; CP: cytoskeletal protein. **b** The immunofluorescence of NLRP7. The co-localized fluorescent signals of NLRP7 and DAPI are observed under a confocal fluorescent microscope. The cytoplasmic juxtanuclear aggregates of NLRP7 are observed in un-decidualized T-HESCs. Intense NLRP7 signals appear in the nucleus after induced decidualization. DAPI was used as the counter stain

We further examined the subcellular location of NLRP7 in the decidualized T-HESCs. The NLRP7 proteins in the cytoplasmic, membrane, nuclear soluble, chromatin-bound and cytoskeletal fractions from the decidualized and non-decidualized T-HESCs were detected by Western blot analysis. The results show that Lamin B appeared in the soluble and chromatin-bound nuclear extracts, and α-tubulin appeared in the cytoplasmic, membrane, and cytoskeletal extracts of the decidualized or non-decidualized T-HESCs (Fig. 4a). In the non-decidualized T-HESCs, most of NLRP7 proteins presented in the membrane and cytoplasmic fractions, and a few of the NLRP7 proteins presented in the soluble nuclear fraction. After in vitro decidualization, the amount of NLRP7 proteins decreased in the membrane and cytoplasmic fractions, while the amount of NLRP7 increased in the soluble nuclear fraction (Fig. 4a). It is noteworthy that only the short isoform was translocated into the nucleus. Under the confocal fluorescence microscope, the immunofluorescence signals of NLRP7 protein were observed to mainly localize in a cytoplasmic juxtanuclear aggregate before the in vitro decidualization. The NLRP7 fluorescent signals diffusely presented in the cytoplasm and in the nucleus of the decidualized T-HESCs, compared to the non-decidualized T-HESCs (Fig. 4b). This finding suggests that NLRP7 proteins distributed diffusely and translocated to the nucleus of T-HESCs after in vitro decidualization.

### NLRP7 promoted PR activity

**Fig. 5:**
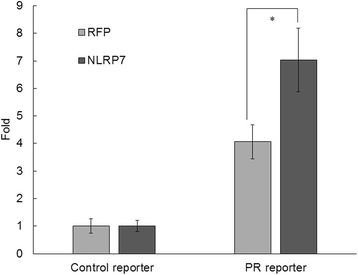
NLRP7 promoted progesterone receptor activity. T-HESCs were co-transfected with PR reporter and NLRP7-expressed vector or RFP-expressed vector. Cells co-transfected with control reporter and NLRP7-expressed vector or RFP-expressed vector served as the control. The transfected cells were subjected to in-vitro decidualization. After 24 h, the firefly and Renilla luminescence were detected. The results are shown as the relative ratio of PR reporter activity to control reporter activity. NLPR7-expressed PR reporter group has higher progesterone receptor activity than that of RFP-expressed group. The statistics were calculated from three individual experiments. *p*-values less than 0.05 are marked with “*”

After ovulation, the progesterone level rises and induces decidualization. Given upregulation and nuclear translocation of NLRP7 in the decidualized T-HESCs, we further used the PR reporter to test whether NLRP7 is involved in the progesterone-induced decidualization. The results show that T-HESCs co-transfected with PR reporter and NLRP7-expessed vector had a higher level of luciferase activity than cells co-transfected with PR reporter and RFP-expressed vector. Cells transfected with the control reporter group showed a very low level of luciferase activity due to the lack of a PR response element (Fig. 5). This finding suggests that NLRP7 contributed to in vitro decidualization of endometrial stromal cells via promoting the progesterone receptor activity.

## Discussion

NLRP7 is a cytosolic protein and has diverse functions in the areas of immunity and reproduction. Given that defective decidualization of the endometrium has been implicated in miscarriage and preeclampsia [24, 25], our immunohistochemistry results show that NLRP7 is expressed in the first trimester endometrium. Therefore, we explored the link between NLRP7 and decidualization of endometrium in this study. A report showed that the level of PRL production, as well as intracellular cAMP levels of endometrial stromal cells, both increased after nine days of progesterone treatment only [30]. Used in combination with cAMP analog treatment, this can sensitize the cells to progesterone and accelerate the decidualization [31]. In this study, we established in vitro decidualization by the treatment of MPA and 8-Br-cAMP to induce the decidualization of T-HESCs in a short time frame [26]. In the human endometrium, the morphologically epitheloid-like changes of stromal cells associated with decidualization are first apparent approximately nine days after ovulation [32]. In this study, we found the epitheloid-like appearance of the decidualized T-HESCs and the NLRP7 expression was up-regulated during the decidualization process. Whether the expression of NLRP7 was regulated by the treatment directly or not awaits further investigation. NLRP7 knockdown suppressed decidualization according to the reduced IGFBP-1 level. Although it could be argued that the reduced IGFBP-1 expression was due to the off-target effect of siRNA, overexpressed NLRP7 also upregulated the IGFBP-1 expression as the stromal cells underwent decidualization. We speculate that while NLRP7 contributes to in vitro decidualization, NLRP7 itself is not sufficient to induce decidualization.

In human pregnancy, progesterone acts via the PRs to regulate the expression of target genes for decidualization [33]. Microarray data showed that decidualization involves reprogramming of gene expression in human endometrial stromal cells [34–37]. Upon progesterone binding, PRs recognize specific response elements located in the promoter region of target genes. Many co-activators or co-repressors are also recruited to the DNA-bound receptors to help modulate gene transcriptions. Besides, PRs can also directly activate multiple signaling pathways to regulate gene expressions [38–40]. In this study, we further found that the overexpressed NLRP7 was able to promote the transcription activity of PR and the native NLRP7 relocalized to the nucleus after in vitro decidualization of endometrial stromal cells. Indeed, some of NLR family members have cytosolic recognition-independent functions. For example, the class II trans-activator, CIITA, was reported to associate with transcription factors and coordinate histone modifications in the promoter to regulate the MHC class II gene expression [41, 42]. The NLR Family CARD Domain Containing 5, NLCR5, was found to bind and trans-activate the promoters of MHC class I genes [43]. NLRP3 was demonstrated to act as a key transcription factor in the Th2 differentiation of CD4+ T cells [44]. A recent study directly showed that the transiently expressed NLRP7 trapped and relocalized the nuclear transcription repressor ZBTB16 to the cytoplasmic juxtanuclear aggregate in HEK293T cells, and diffusely colocalized with the transient expressed KHDC3L in the cytoplasm and in the juxtanuclear aggregates [45]. It is plausible to speculate that NLRP7 could be a transcription cofactor of PR and involved in the decidualization process. However, further study is needed to explore whether and how NLRP7 is involved in the decidualization process.

Alternative splicing creates different NLRP7 protein isoforms. There are six *NRLP7* transcript isoforms. Of these, five differ in the numbers of the C-terminal LRR domains and have around 963–1065 amino acids (MW ~ 105–118 kDa), and one isoform lacks the central NACHT domain and has 672 amino acids (MW ~74 kDa) [29]. In the in vitro decidualization model, two NLRP7 protein isoforms were dominantly detected. The small one with a molecular weight close to 100 kDa seemed to be important for decidualization considering only this isoform was translocated into the nucleus. NLRP7 siRNA also only down-regulated the small isoform, and interfered with the decidualization process, while preserving the large isoform. Because the LRR domain of NLRP7 is responsible for the recognition of pathogen-associated molecular patterns (PAMPs) and damage-associated molecular patterns (DAMPs) or protein-protein interaction [46, 47], the two isoforms that differ in the number of LRR domains may have distinct functions in the endometrial stromal cells. Data supporting the aforementioned hypothesis comes from two previous studies, in which NLRP7 proteins carrying mutations in the LRR regions caused dysregulated IL-1β expression in the LPS-stimulated monocytes or reconstituted NLRP7 inflammasome [3, 5]. The target site of NLRP7 siRNA was located in the N-terminal PYD domain in order to suppress all isoforms. However, only the small form was affected by siRNA in this study. It was thus suggested that the secondary structure of RNAs or RNA binding proteins may interfere with an siRNA’s efficacy [48]. Different *NLRP7* transcript isoforms may present with distinctive 3D structures to respond to small interfering RNA. Many mutations of NLRP7 have been reported in women with recurrent HM or RM [10, 49]. However, the inflammasome functions of NLRP7 mutations and their effects on reproduction are not clear. Considering the canonical role of NLRP7 in immunoregulation, loss-of-function mutations of the NLRP7 gene could enable HM patients to tolerate growth of moles due to impaired immune surveillance [5]. On the other hand, gain-of-function mutations of NLRP7 genes may render patients hyperactive to infections and lead to excessive or auto-inflammation [3]. The inflammasome effects of the NLRP7 and the different isoforms of NLRP7 on the decidualization deserve further investigation.

## Conclusion

NLRP7 was upregulated and translocated to the nucleus of the endometrial stromal cells in an in vitro decidualization model. Overexpressed NLRP7 promoted IGFBP-1 expression and PR reporter activation. IGFBP-1 expression decreased with the knockdown of NLRP7. We provide the first clue that NLRP7 may contribute to the decidualization of endometrial stromal cells. Given defective decidualization has been implicated in miscarriage or preeclampsia, our finding may provide a new avenue to elucidate the mechanism of endometrial dysfunction as well as female reproductive failure.

## Abbreviations

8-Br-cAMP: 8-Bromoadenosine 3':5'-cyclic monophosphate
AEC: 3-Amino-9-ethylcarbazole
DAMPs: Damage-associated molecular patterns
GAPDH: Glyceraldehyde 3-phosphate dehydrogenase
HM: Hydatidiform mole
hTERT: Human telomerase reverse transcriptase
IGFBP-1: Insulin-like growth factor-binding protein 1
LRR: Leucine rich repeat
LV-NLRP7: NLRP7-expressed lentivirus
LV-RFP: RFP-expressed lentivirus
MPA: Medroxyprogesterone acetate
NACHT: Nucleotide-binding oligomerization domain
PAMPs: Pathogen-associated molecular patterns
PR: progesterone receptor
PRL: Prolactin
PYD: Pyrin domain
RM: Recurrent miscarriage
TBST: Tris-buffered saline, 0.05% Tween 20
T-HESCs: hTERT-immortalized human endometrial stromal cells
